# The Gut Microbiota in Perimenopausal Anxiety: A Novel Therapeutic Pathway Through Diet

**DOI:** 10.3390/nu18050743

**Published:** 2026-02-26

**Authors:** Giuseppe Marano, Claudia d’Abate, Ilaria Ianes, Giuseppe Sorrenti, Gianandrea Traversi, Rosanna Esposito, Francesco Pavese, Tatiana D’Angelo, Paola Fuso, Gianluca Franceschini, Ida Paris, Marianna Mazza

**Affiliations:** 1 Department of Neuroscience, Head-Neck and Chest, Section of Psychiatry, Fondazione Policlinico Universitario A. Gemelli IRCCS, Largo Agostino Gemelli 8, 00168 Rome, Italy; 2 Department of Neuroscience, Section of Psychiatry, Università Cattolica del Sacro Cuore, 00168 Rome, Italy; 3 Department of Molecular and Developmental Medicine, Obstetrics and Gynecological Clinic, University of Siena, 53100 Siena, Italy; 4 Department of Gynecology, San Carlo Nancy Hospital, 00165 Rome, Italy; 5 Unit of Medical Genetics, Department of Laboratory Medicine, Ospedale Isola Tiberina-Gemelli Isola, 00186 Rome, Italy; gianandrea.traversi@gmail.com; 6 Unit of Gynecology and Obstetrics, Ospedale San Giuseppe Moscati, 81031 Aversa, Italy; 7 Division of Gynecologic Oncology, Department of Woman and Child Health and Public Health, Fondazione Policlinico Universitario A. Gemelli IRCCS, 00168 Rome, Italy; 8 Breast Surgery Unit, Department of Woman and Child’s Health and Public Health Sciences, Fondazione Policlinico Universitario A. Gemelli IRCCS, 00168 Rome, Italy; 9 Department of Medical and Surgical Sciences, Catholic University of the Sacred Heart, 00168 Rome, Italy

**Keywords:** gut microbiota, menopause, anxiety, mental health, neuroinflammation, nutrition

## Abstract

**Background:** Perimenopause is characterized by pronounced fluctuations in ovarian steroids, which are associated with an increase vulnerability to anxiety symptoms. Growing evidence indicates that declining estrogen levels influence gut microbiota composition and microbial metabolic activity, thereby modulating neuroimmune and neuroendocrine pathways involved in emotional regulation. This review explores gut microbiota alterations occurring during the menopausal transition and critically evaluates dietary strategies targeting microbiota–gut–brain mechanisms potentially relevant to perimenopausal anxiety. **Methods:** A structured literature search was conducted in PubMed, Scopus, and Web of Science to identify clinical, translational, and preclinical studies addressing: (i) gut microbiota changes across perimenopause and menopause; (ii) microbiota–gut–brain pathways implicated in anxiety; and (iii) dietary patterns, nutrients, probiotics, and prebiotics with documented microbiota-modulating effects. The available evidence was synthesized narratively, with particular attention to biological plausibility and clinical relevance. **Results:** The perimenopause transition is associated with reduced microbial diversity, depletion of *Lactobacillus*, *Bifidobacterium*, and short-chain fatty acid (SCFA)-producing taxa, and enrichment of pro-inflammatory microbial signatures. These alterations are linked to increased intestinal permeability, altered tryptophan-kynurenine metabolism, immune activation, and dysregulated hypothalamic–pituitary–adrenal axis activity. Dietary interventions, including Mediterranean-style diets, fiber- and polyphenol-rich foods, fermented products, and selected probiotic and prebiotic formulations, have been shown to modulate gut microbial composition, enhance SCFA production, and attenuate inflammatory and neuroendocrine stress pathways. Preliminary evidence suggests potential anxiolytic benefits; however, randomized controlled trials specifically targeting perimenopausal populations remain limited. **Conclusions:** Gut microbiota dysbiosis may contribute to anxiety vulnerability in perimenopausal women through interconnected immune, metabolic, and neuroendocrine mechanisms. Dietary modulation of the intestinal microbiota represents a biologically plausible and low-risk complementary approach to support emotional well-being during this transitional period. Well-designed, perimenopause-specific clinical trials are needed to confirm efficacy and inform microbiome-based nutritional strategies.

## 1. Introduction

During perimenopause, estrogen levels decline significantly, leading to changes that affect not only female physiology but also psychological well-being [1]. Somatic symptoms, such as hot flashes and sweating, sleep disturbances, urinary and genital infections, and musculoskeletal pain, frequently coexist with affective manifestation, including anxiety and depressive symptoms, resulting in a significant deterioration of quality of life [2].

In parallel, the menopausal transition marks the onset of age-related conditions such as osteoporosis, cardiovascular disease, and neurodegenerative disorders, all of which are closely linked to overall physical and mental health. Given the increasing life expectancy of women worldwide, health issues affecting menopausal women are drawing growing attention [3]. Mental health is defined as a state in which an individual is able to realize their abilities, cope effectively with daily stressors, work productively, and contribute meaningfully to their community [4]. Disruptions in mental health are typically characterized by abnormal thinking patterns, emotional dysregulation, altered perception, and changes in behaviour and interpersonal relationships [5]. Epidemiological data indicate a substantial and rising burden of mental disorders, particularly in Western countries. In the United States alone, approximately 46.6 million adults, corresponding to 14.7% of the population, reported having a mental health disorder in 2017, with anxiety and depressive disorders representing the most prevalent conditions across demographic groups [5]. Focusing on the health of women during the perimenopausal period can improve overall quality of life by alleviating both physical and psychological symptoms, while also contributing to more sustainable healthcare costs [6]. Currently, menopausal hormone therapy (MHT) remains the most widely used and effective treatment for alleviating menopausal symptoms and preventing age-related diseases, largely due to the protective effects of estrogen [7].

However, the decline in estrogen also influences the vaginal and intestinal microbiota, contributing to mucosal atrophy, reduced glycogen content, and decreased *Lactobacillus* abundance. These ecological shifts underlie the genitourinary syndrome of menopause (GSM) and increase susceptibility to urogenital and reproductive infections. Estrogen also modulates the intestinal, urethral, and oral microbiomes, where microbial dysbiosis has been associated with several pathologies [7]. Unfortunately, systematic investigations into vaginal microbiological changes during estrogen deficiency or under MHT remain limited. Despite its benefits, MHT carries potential risks, such as thromboembolic events and the possible progression of undiagnosed breast lesions, which have raised concerns among both physicians and patients.

To improve women’s health during menopause, complementary approaches are gaining increasing attention. From a nutritional perspective, compounds such as polyphenols, dietary fibers, soy isoflavones, and bioactive components in fermented soy-based foods have demonstrated positive effects on cardiovascular health, gut function, bone metabolism, and the alleviation of menopausal symptoms [7]. Moreover, probiotic supplementation has been shown to beneficially influence vaginal and intestinal microbial communities in postmenopausal women and to help mitigate estrogen deficiency-related symptoms [8]. Herbal extracts, such as soy extract, black cohosh (Cimicifuga racemosa), and red clover, along with traditional therapies like acupuncture and moxibustion, represent additional supportive options for promoting well-being during this life stage by modulating endocrine, nervous, and immune system functions [9].

Emerging evidence is increasingly linking psychiatric disorders to nutrient intake and to the individual composition and function of the human microbiome, which plays a fundamental role in immune regulation and intestinal health [5]. Adequate nutrition is essential for the synthesis of neurotransmitters such as serotonin, dopamine, and norepinephrine, which regulate mood, appetite, and cognition. Their production depends on the intake of key nutrients including tryptophan, vitamin B6, vitamin B12, folate, phenylalanine, tyrosine, histidine, choline, and glutamic acid. Furthermore, omega-3 fatty acids are critical for dopaminergic and serotonergic neurotransmission, influencing both depression and anxiety [10]. A poor-quality diet with insufficient nutrient intake can contribute to the onset of mental and behavioral disorders. For this reason, nutritional interventions are being explored as promising strategies for prevention and treatment [11]. The International Society for Nutritional Psychiatry Research (ISNPR) has also emphasized the need to integrate dietary approaches into psychiatric care [5]. Additionally, the immune system, socioeconomic context, and lifestyle factors have a considerable impact on the overall health of menopausal women.

Considering all these aspects, the aim of this narrative review is to critically analyze the role of gut microbiota dysbiosis in perimenopausal anxiety, and to evaluate nutritional strategies as complementary and supportive therapies during this crucial stage of women’s lives. This review is distinguished by its specific focus on perimenopause as a biological stage that is often underrepresented in anxiety-related research, and by its integrative analysis of the interactions among estrogen fluctuations, gut microbiota dysbiosis, and gut–brain axis mechanisms, with the aim of exploring the potential role of nutrition as a complementary and non-prescriptive strategy.

## 2. Materials and Methods

A structured literature search was conducted in MEDLINE and Scopus to identify English-language publications relevant to the relationship between gut microbiota, anxiety symptoms during the perimenopausal transition, and dietary modulation, from database inception through October 2025. The search strategy combined selected keywords and Medical Subject Headings (MeSH), including “Gut Microbiota,” “Microbiome,” “Perimenopause,” “Menopause,” “Anxiety,” “Mental Health,” “Neuroinflammation,” “Diet,” “Nutrition,” “Probiotics,” “Prebiotics,” “Fiber,” and “Psychobiotics,” with the aim of capturing both clinical and mechanistic contributions to the topic rather than exhaustively identifying all available evidence.

This narrative review included original research articles—such as randomized and non-randomized controlled trials, prospective and retrospective cohort studies, and case–control studies—as well as selected review articles considered informative for contextualization. Studies were eligible if they examined associations between gut microbiota alterations and anxiety-related outcomes during the perimenopausal period, or if they provided relevant mechanistic or clinical insights applicable to this transition. Evidence derived from non-perimenopausal populations or preclinical models was included to inform biological plausibility and was interpreted as indirect.

Titles and abstracts were screened by two reviewers according to predefined inclusion criteria, with full-text evaluation performed when appropriate. The narrative synthesis addresses key physiological, clinical, and mechanistic aspects of the topic, beginning with hormonal and metabolic changes associated with perimenopause, followed by an overview of the gut–brain–microbiota axis, evidence linking microbial dysbiosis with anxiety symptoms, and emerging dietary strategies aimed at modulating microbial composition and function.

**Figure 1:**
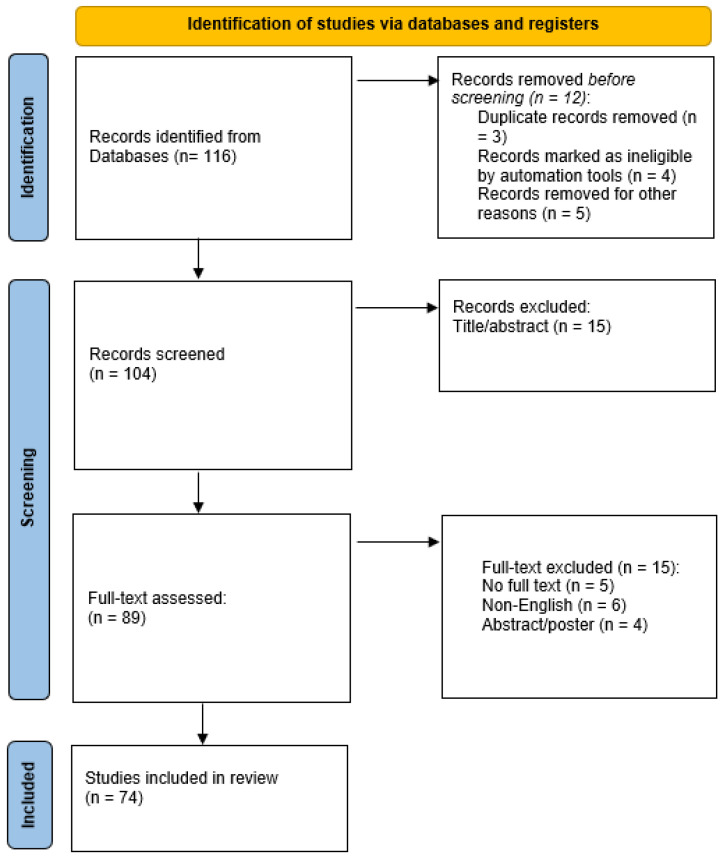
PRISMA flow diagram of the study selection process.

The study selection process was informed by PRISMA principles solely as a reporting framework to transparently document the identification and screening of the literature. PRISMA was used to describe inclusion criteria, duplicate management, and selection flow, without implying adherence to systematic review methodology or the application of formal risk-of-bias assessment tools. The selection process yielded 116 records, of which 74 were ultimately included in the narrative synthesis, as summarized in Figure 1.

The selection process is illustrated in Figure 1, which summarizes the identification, screening, eligibility, and inclusion phases. Consistent with the narrative nature of this review, the literature search was structured but not exhaustive, and the synthesis aimed to provide an integrative and conceptual overview of the existing evidence rather than a formal evaluation of intervention effectiveness. Accordingly, no standardized risk-of-bias assessment tools or quantitative quality scoring systems were applied.

## 3. Pathophysiology of Anxiety in Perimenopause

Menopause marks the permanent cessation of reproductive function and is accompanied by profound changes in the female hormonal milieu. Throughout the perimenopausal interval, ovarian steroid secretion becomes increasingly erratic, particularly with respect to estradiol, and this unstable phase may extend for more than five years before consistently low estradiol levels are established. Following menopause, estradiol concentrations remain chronically reduced, whereas circulating follicle-stimulating hormone (FSH) and luteinizing hormone (LH) increase substantially [12]. Hormonal fluctuations occurring across the menopausal transition exert well-documented effects on central nervous system (CNS) structure and function, with important implications for affective regulation and emotional stability.

The perimenopausal transition unfolds through a sequence of neuroendocrine phases. Initially, the hypothalamic-pituitary-gonadal (HPG) axis is still capable of compensatory adjustments that preserve reproductive function and endocrine output. As ovarian follicular depletion progresses, steroidogenic capacity diminishes, culminating in the final menstrual period and, subsequently, a sustained state of low ovarian hormone secretion. Under physiological conditions, antral follicles secrete inhibin B, which suppresses FSH release and facilitates follicular recruitment and maturation [13]. The progressive reduction in inhibin B associated with ovarian aging results in a gradual rise in circulating FSH concentrations [14]. Concomitantly, ovulatory cycles become increasingly irregular, and the pulsatile activity of gonadotropin-releasing hormone (GnRH) accelerates [15]. Regulation of GnRH pulsatility depends on a hypothalamic network composed of stimulatory neuropeptides—neurokinin B (NKB) and kisspeptin (Kp)—and the inhibitory peptide dynorphin (DYN) [16]. This network, commonly referred to as the GnRH pulse generator, is principally located in the arcuate nucleus and is tightly modulated by circulating estrogen and progesterone [17]. Although follicular attrition is a central driver of these changes, experimental models suggest that aging-related alterations within the hypothalamus itself may independently contribute to disrupted GnRH signaling, with kisspeptin playing a key mechanistic role [18].

During the late menopausal transition, reduced estrogen-mediated negative feedback prolongs the half-life of LH and FSH, leading to additional elevations in their serum concentrations, up to fifteen-fold for FSH and ten-fold for LH in the postmenopausal period [15]. The coordinated behaviour of KNDy neurons and the pulsatile release of Kp are fundamental for GnRH pulse generation, although other neurotransmitter systems also regulate this process. Excitatory glutamatergic input supports the initiation and synchronization of KNDy activity, whereas inhibitory γ-aminobutyric acid (GABA) modulates pulse frequency [19]. With advancing age, glutamatergic tone declines while GABAergic activity increases within the hypothalamus, further altering GnRH rhythmicity [20]. Additionally, the loss of negative steroid feedback alters kisspeptin expression, a mechanism thought to play a role in the development of vasomotor symptoms (VMS) during menopause [21].

Age-related shifts in neurotransmitter dynamics, synaptic signalling, and hormone-receptor expression disrupt the neurobiological equilibrium characteristic of the reproductive years and are believed to contribute to menopausal symptoms such as mood disturbances, vasomotor instability, and depressive features [22]. Large cohort studies support this association: in the Study of Women’s Health Across the Nation (SWAN), women in early or late perimenopause and in postmenopause reported significantly higher scores on the Center for Epidemiological Studies Depression Scale (CES-D), describing symptoms such as low mood, anhedonia, appetite and concentration difficulties, fatigue, guilt, restlessness, and suicidal ideation [23]. Anxiety symptoms also tend to intensify during the menopausal transition, regardless of baseline anxiety levels [24].

These neuroendocrine mechanisms interact with a broad range of psychosocial factors. Pre-existing mental health vulnerabilities, socioeconomic stressors, and interpersonal challenges may amplify sensitivity to hormonal fluctuations. In addition, menopause-related changes in sleep quality, body image, sexual function, and overall physical health can further influence emotional resilience and stress responsivity [23]. Providing clear, evidence-based information about the physiological and psychological modifications occurring during this period is crucial to support women in navigating the menopausal transition [25,26,27].

## 4. The Gut Microbiota and the Microbiota–Gut–Brain Axis: Mechanisms Underlying Anxiety

The influence of the microbiota on human health and disease is now widely recognized, and the microbiota–gut–brain axis has emerged as a major frontier with important implications for understanding human physiology [28]. Humans coexist with trillions of microorganisms, including bacteria, viruses, and fungi, that inhabit all body districts and participate in maintaining health or driving disease states [29]. In particular, the term intestinal microbiome refers to the collective genomes of all microorganisms residing in the gut [30]. Advances in sequencing technologies have enabled deeper exploration of microbial communities, highlighting their crucial role in shaping host health. The intestinal microbiota, especially the bacterial component, has gained increasing attention for its impact on immune maturation, neuroinflammation, and neurobehavioral profiles, moving beyond traditional measures of microbial diversity [31,32].

Innate and adaptive immune systems represent critical interfaces between the gut microbiota and the CNS. Innate immunity provides the first line of host defense by integrating microbial signals to coordinate local and systemic immune responses, while adaptive immunity confers specificity and immunological memory. Together, these immune pathways constitute key mediators of microbiota-driven effects on brain function and behaviour [33]. Communication along the microbiota–gut–brain axes occurs through multiple routes, involving both neural circuits and chemical messengers, although the precise mechanisms underlying these interactions remain only partially elucidated. The autonomic nervous system (ANS), through its sympathetic and parasympathetic divisions, interfaces with the immune system and plays a fundamental, involuntary role in maintaining homeostasis. It supports bidirectional communication along the microbiota–gut–brain axis, shaping gastrointestinal function and responding to environmental stimuli through feedback circuits [28].

The vagus nerve, together with pelvic afferent pathways, represents a central hub in this network by providing a direct anatomical link between the gut and the brain. These fibers collect information from the intestinal tract and modulate gastrointestinal and immune functions through sympathetic/splanchnic and parasympathetic efferents, influencing emotional and behavioral responses [34]. The enteric nervous system (ENS), often referred to as the “third branch” of the ANS [35], forms an intrinsic neural network within the gastrointestinal tract and interacts closely with intestinal immune populations, including macrophages, T cells, and innate lymphoid cells. The gut microbiota shapes both ENS development and function: germ-free models show marked ENS immaturity [36] and immune dysfunction, demonstrating the essential role of microbes in neuroimmune development [37]. Antibiotics and dietary factors can also modify ENS architecture and immune activity, altering intestinal motility and secretion.

The microbiota triggers the release of cytokines, chemokines, neurotransmitters, neuropeptides, hormones, and microbial metabolites, which enter the bloodstream and lymphatic system or modulate neural signals transmitted by vagal and spinal afferents. These signals constantly inform the brain about the intestinal environment and influence a broad range of physiological and behavioral functions [38].

Recent evidence has revealed a “shared chemical language” between host and microbes: numerous bacterial species produce or respond to molecules such as serotonin (5-HT), GABA, catecholamines, and indole derivatives, thereby influencing mood, cognition, and immune responses [39,40]. Tryptophan metabolism, shared by microbes and host, produces serotonin and kynurenine, shaping gastrointestinal serotonergic systems, immune regulation, and mental health [41]. Microbial metabolites derived from tryptophan, particularly indoles, affect intestinal permeability, inflammation, and metabolic pathways, with production modulated by stress and circadian rhythms [42].

Crucial neurotransmitters involved in anxiety regulation, GABA, dopamine (DA), norepinephrine (NE), and serotonin, can cross the blood–brain barrier under specific conditions such as inflammation, influencing brain regions that mediate anxiety-related responses [43]. GABA, the main inhibitory neurotransmitter in the CNS, plays a pivotal role in emotional and physiological regulation. *Bifidobacterium* and *Lactobacillus* species can produce GABA through fermentation. Preclinical evidence suggests that GABAergic signaling may interact with BDNF–TrkB pathways, potentially influencing neuroplasticity, supporting hippocampal function and potentially mitigating stress-induced anxiety and depression by also increasing 5-HT and DA levels; however, these mechanisms have been primarily described in experimental models and cannot yet be directly extrapolated to perimenopausal anxiety in humans [44].

Serotonin modulates emotional responses and stress reactivity by acting on neurons in the amygdala and prefrontal cortex. Most of the mechanistic insights into these processes derive from preclinical models and adult human cohorts not specifically focused on the perimenopausal transition. Approximately 95% of the body’s serotonin is synthesized in the gut by enterochromaffin cells [45], which use microbial components, cellular debris, and tryptophan. Bacterial genera such as *Enterococcus*, *Escherichia*, and *Lactobacillus* can also produce 5-HT from tryptophan. Dopamine, a key regulator of reward, motivation, and attention, is modulated by several gut bacteria including *Bacillus*, *Lactobacillus*, and *Enterococcus* [46]. Evidence for these interactions largely stems from preclinical investigations and observational human studies not specific to perimenopause. *Enterococcus faecium* produces L-DOPA, which crosses the blood–brain barrier and is converted into dopamine in dopaminergic terminals [47]. *Bacillus licheniformis* can reduce tryptophan, DA, and GABA levels by altering gut conditions and increasing SCFA production, an effect associated with anxiolytic-like behaviors in animal models [48]. A newly described mechanism involves microbial conversion of 3-methoxytyramine (3MT) into dopamine after host COMT-mediated methylation, although its relevance in human anxiety during perimenopause remains to be established [49]. Norepinephrine, another key neurotransmitter in anxiety, is influenced by its precursors and by specific microbes. Heat-inactivated *Enterococcus faecalis* increases β3-adrenergic receptor activity in the prefrontal cortex, boosting NE release, whereas *Bifidobacterium* CECT 7765 reduces NE levels in the hypothalamus [50].

The cholinergic system also participates in emotional regulation. Glutamate, the main excitatory neurotransmitter, is deeply involved in anxiety disorders. Some bacteria (*Bifidobacterium*, *Lactobacillus*) convert glutamate into GABA, indirectly influencing anxiety and emotional processing, with evidence mainly coming from preclinical and non-perimenopause-specific human studies [51]. Glutamate-NMDA receptor interactions regulate neuronal excitability and are central to anxiety pathophysiology [52].

In summary, the microbiota–gut–brain axis represents a multidirectional system in which neural, endocrine, immune, and metabolic pathways converge. Disruptions in intestinal homeostasis may increase permeability, activate systemic inflammation, and influence brain circuits involved in emotional regulation; these mechanisms are *hypothesized* to be particularly relevant during hormonally dynamic stages such as perimenopause [46], although direct perimenopause-specific human evidence remains limited. These neural, endocrine, and immune components of the microbiota–gut–brain axis (MGBA) operate in a highly integrated manner and offer an important framework for understanding behavioral and mood disorders, particularly anxiety-related conditions.

## 5. Gut Microbiota Alterations During Perimenopause

The perimenopausal transition represents a sensitive endocrine and metabolic period marked by fluctuating estrogen levels, progressive ovarian senescence, and a shift toward a pro-inflammatory systemic milieu. Although these physiological changes are well characterized, their effects on the intestinal microbiota are primarily inferred from observational human studies and supported by mechanistic data from preclinical models.

These physiological changes directly influence the structure and function of the intestinal microbiota, resulting in reproducible alterations detectable across multi-omics platforms. Recent longitudinal and cross-sectional studies provide converging evidence that women transitioning through perimenopause exhibit marked reductions in α-diversity and altered β-diversity profiles, reflecting both a loss of microbial richness and a remodeling of community composition. Importantly, these alterations correlate with symptom severity, metabolic risk clusters, and inflammatory markers, suggesting a bidirectional relationship between hormonal transitions and microbial ecology [53,54].

Declining estrogen levels exert a central influence on these ecological changes. Large cohort analyses demonstrate that menopausal transition is associated with reduced microbial diversity, enrichment of *Bacteroides* and *Prevotella*, and depletion of beneficial taxa such as *Akkermansia muciniphila*. A central mechanistic link between estrogen decline and microbial disruption is mediated through the estrobolome, the subset of microbial genes capable of metabolizing estrogens and regulating enterohepatic recirculation. Dysbiosis-associated reductions in β-glucuronidase activity impair estrogen deconjugation and reabsorption, thereby amplifying estrogen deficiency and its downstream metabolic and neuropsychiatric consequences [55]. Support for this mechanism is largely derived from experimental and animal studies.

Preclinical models reinforce this concept: ovariectomized rodents demonstrate rapid dysbiosis, increased gut permeability, elevated IL-6, TNF-α, and IL-1β, and behavioral changes that partially normalize with estrogen replacement or fecal microbiota transplantation from estrogen-replete donors [56]. These findings highlight the dynamic interplay between ovarian hormones, microbial metabolism, and mucosal immune regulation, though direct confirmation in perimenopausal human populations remains limited.

Consistent findings include reductions in *Bifidobacterium* and *Lactobacillus* species, accompanied by decreases in butyrate-producing genera such as *Faecalibacterium prausnitzii* and *Roseburia* spp. Reductions in butyrate producers weaken colonic barrier integrity, facilitate lipopolysaccharide (LPS) translocation, and promote systemic low-grade inflammation, a mechanism increasingly linked to cardiometabolic alterations and changes in mood regulation. These microbial shifts parallel rising visceral adiposity, deteriorating glucose tolerance, and increased inflammatory cytokines commonly observed during perimenopause [54,57] although causality cannot be inferred from these associations alone.

Emerging findings connect these mechanisms to anxiety and affective disturbances. Women with perimenopausal panic disorder exhibit reductions in SCFA-producing taxa and increases in *Bacteroides*, with microbial shifts correlating with established clinical severity scores [58]. Reviews on the microbiota–gut–brain axis propose that dysbiosis-driven inflammation, alterations in neurotransmitter pathways, and disruption of microbially derived metabolites may exacerbate affective symptoms during hormone transition states [59]. Preclinical models further support a causal role: sex hormone withdrawal induces anxiety- and depression-like behaviors through microbiota-dependent mechanisms, and restoring beneficial taxa- particularly *Lactobacillus* spp.-mitigates these effects, whereas microbiota depletion eliminates this behavioral rescue [60].

Dietary interventions represent one of the most accessible and impactful levers for modifying gut microbial composition during the menopausal transition. Seed-enriched, fiber-rich, plant-based dietary patterns have been shown to shift key microbial taxa, including *Verrucomicrobia* and *Synergistetes*, and simultaneously improve cognitive performance and neural functional connectivity, underscoring the interconnectedness of diet, microbiota, and brain function [61]. A comprehensive meta-analysis confirmed that microbiota-targeted interventions, including probiotics, prebiotics, and synbiotics, significantly reduce anxiety and depressive symptoms in women experiencing dynamic hormonal fluctuations, reinforcing the potential for diet-microbiota strategies as complementary therapeutic tools [62], while acknowledging heterogeneity in study populations and outcome measures.

## 6. Dietary Interventions Targeting Gut Microbiota to Mitigate Perimenopausal Anxiety

During perimenopause, alterations in gut microbiota composition and function may contribute to heightened vulnerability to anxiety. Dietary strategies capable of restoring microbial diversity and modulating neuroimmune and neuroendocrine pathways represent a promising, non-pharmacological approach. Evidence indicates that specific dietary patterns, nutrients, and microbiota-targeted interventions influence emotional regulation through mechanisms involving the microbiota–gut–brain axis [63]. At the current state of knowledge, however, these findings do not yet allow the definition of standardized, protocol-driven dietary interventions specifically targeting perimenopausal anxiety.

### 6.1. Dietary Patterns and Whole-Food Approaches

Dietary patterns exert broad effects on intestinal microbial communities. Adherence to the Mediterranean diet (MD), characterized by high intake of fruits, vegetables, legumes, whole grains, nuts, and extra-virgin olive oil, is associated with increased microbial richness and abundance of SCFA producers, alongside reductions in pro-inflammatory taxa [64]. These microbial changes contribute to enhanced epithelial barrier integrity, reduced systemic inflammation, and improved regulation of stress-related neuroendocrine pathways, mechanisms that may mitigate anxiety symptoms during perimenopause [65]. It should be noted that evidence supporting these effects in perimenopausal women is largely indirect and not derived from perimenopause-specific randomized controlled trials. Fermented foods, including yogurt, kefir, kimchi, sauerkraut, and miso, introduce live microorganisms and bioactive metabolites that enhance microbial diversity and reduce inflammatory cytokines. Controlled feeding trials demonstrate that fermented-food consumption increases gut microbial diversity and lowers circulating markers of inflammation, supporting their potential role in promoting emotional resilience [66]. Nevertheless, the optimal type, quantity, and duration of fermented food intake for anxiety modulation during perimenopause remain to be established.

In this context, a recent pilot study by Guo et al. in perimenopausal women demonstrated that a plant-based dietary intervention rich in seed-containing vegetables was associated with changes in gut microbiota composition and intrinsic brain activity, as well as improvements in selected cognitive domains, providing a preliminary and population-specific example of diet–microbiota–brain interactions during the menopausal transition [61].

Overall, while these dietary patterns provide biologically plausible support for modulating gut–brain axis-related pathways, current evidence does not yet allow firm conclusions regarding their direct clinical efficacy on perimenopausal anxiety, underscoring the need for targeted randomized controlled trials.

### 6.2. Bioactive Nutrients and Phytochemicals

Several bioactive dietary components influence microbiota composition and neuroimmune function. Polyphenols, such as resveratrol, catechins, and quercetin, modulate microbial populations, promote beneficial taxa, and reduce neuroinflammatory signaling [67]. Soy isoflavones, including genistein and daidzein, act as phytoestrogens and are metabolized by gut bacteria into equol, a compound with estrogenic and anti-inflammatory properties relevant to emotional regulation during estrogen decline [68]. Interindividual variability in microbial metabolism of these compounds further limits the formulation of uniform clinical recommendations. Lignans in flaxseed are converted into enterolignans with antioxidant and estrogen-modifying activity. Omega-3 fatty acids improve microbial diversity, reduce neuroinflammation, and modulate neurotransmission. Vitamin D, commonly deficient during menopause, supports mucosal immunity and influences microbial composition, with deficiency associated with anxiety and depressive symptoms [69]. However, available data are insufficient to define precise dosing regimens or treatment durations specific to perimenopausal anxiety.

### 6.3. Probiotics, Prebiotics, and Synbiotics

Prebiotics (e.g., galacto-oligosaccharides, fructo-oligosaccharides, inulin), probiotics (specific live strains with psychobiotic potential), synbiotics (combinations thereof) can enhance microbial diversity, increase SCFA-producing taxa and reduce systemic inflammation. An overview of microbial-targeted nutritional interventions and their associated gut–brain mechanisms is presented in Table 1. Recent evidence shows that these interventions are capable of modulating key pathways implicated in perimenopausal anxiety, including HPA-axis reactivity, serotonergic signalling, intestinal permeability, and microglial activation. Despite these mechanistic insights, heterogeneity in study design and populations currently precludes the definition of structured clinical pathways or standardized intervention protocols.

A meta-analysis by Dubois et al. [62] demonstrated a statistically significant reduction in anxiety symptoms in women undergoing microbiota-targeted interventions during hormonally dynamic phases, with a standardized mean difference (SMD) of −0.997 (95% CI: −1.684 to −0.311; *p* = 0.004). In addition, Liaquat et al. [70] highlight how modulation of the gut microbiota through probiotics and prebiotics may influence estrogen dynamics and contribute to the management of menopause-related disorders.

Although no randomized controlled trials (RCTs) have yet focused specifically on perimenopausal anxiety, several probiotic strains have demonstrated anxiolytic effects in adult female cohorts and in preclinical models that mimic estrogen-withdrawal physiology. These findings should therefore be interpreted as supportive, hypothesis-generating evidence rather than as a basis for definitive clinical recommendations.

Among the most extensively studied formulations, the combination of *Lactobacillus helveticus* R0052 and *Bifidobacterium longum* R0175 has shown significant reductions in psychological distress and cortisol, together with improvements in intestinal barrier integrity and attenuation of systemic inflammation [71].

Additional psychobiotic candidates include *Lactobacillus plantarum* PS128, which has demonstrated improvements in anxiety and mood outcomes in both clinical and preclinical settings, partly by increasing central dopamine and serotonin availability. Similarly, *Bifidobacterium breve* CCFM1025 has exhibited anxiolytic and antidepressant properties through enhanced SCFA production and downregulation of neuroinflammatory pathways [72]. *Lactobacillus casei* Shirota, a widely studied strain, has also been associated with reductions in anxiety and stress-induced inflammatory markers, reinforcing its relevance in stress-related disorders [71].

Together, these strains share mechanistic actions that are highly pertinent to perimenopausal physiology, including enhancement of butyrate production, stabilization of epithelial barrier function, modulation of immune signalling and attenuation of low-grade inflammation, pathways that may contribute to improved anxiety regulation during the menopausal transition.

Emerging evidence also highlights a role for specific prebiotic substrates in modulating anxiety-related pathways. As with probiotics, data on optimal dosing, duration, and long-term safety in perimenopausal populations remain limited.

Galacto-oligosaccharides (GOS) have been shown to reduce anxiety in young women with high trait anxiety while markedly increasing the abundance of *Bifidobacterium* spp. [71]. Fructo-oligosaccharides (FOS) similarly enhance SCFA-producing taxa and promote microbial diversity, suggesting a potential contribution to emotional regulation. Inulin, a fermentable dietary fibre, increases levels of *Akkermansia muciniphila* and *Bifidobacterium*, two taxa consistently reported as reduced during the menopausal transition [73,74]. Finally, resistant starch (RS) has demonstrated a strong capacity to increase butyrate-producing genera such as *Faecalibacterium* and *Roseburia*, which exert recognized anti-inflammatory and neuromodulatory effects relevant to anxiety pathways [72].

**Table 1:** Probiotic and prebiotic interventions with evidence for gut–brain modulation relevant to anxiety.

Category	Strain/Compound	Key Mechanisms	Study Type/Population	Evidence Summary	Ref.
Probiotic (psychobiotic combination)	*Lactobacillus helveticus* R0052 + *Bifidobacterium longum* R0175	Reduces cortisol levels; improves intestinal barrier integrity; modulates systemic inflammation	Human clinical study (adults, non-menopausal)	Associated with reduced psychological distress and stress-related symptoms; no perimenopause-specific data available	[71]
Probiotic (psychobiotic)	*Lactobacillus plantarum* PS128	Modulates dopamine and serotonin signaling; reduces neuroinflammation	Animal model	Demonstrates anxiolytic effects in preclinical anxiety-like behavior models	[47]
Probiotic (SCFA-enhancing)	*Bifidobacterium breve*	Increases short-chain fatty acids (particularly butyrate); attenuates neuroinflammatory pathways	Preclinical studies (animal/in vitro)	Exhibits anxiolytic and antidepressant-like effects in experimental models	[50]
Probiotic (stress-modulating)	*Lactobacillus casei* Shirota	Modulates stress-induced inflammation; influences gut–brain signaling	Human observational/interventional studies (adults)	Associated with reduced anxiety or stress-related symptoms; menopausal status not specified	[10]
Prebiotic (GOS)	Galacto-oligosaccharides (GOS)	Selectively increases *Bifidobacterium*; modulates HPA axis activity	Human RCT (women with high trait anxiety)	Reduces anxiety-related outcomes in women with elevated baseline anxiety; not specific to perimenopause	[71]
Prebiotic (fermentable fiber)	Inulin	Increases *Akkermansia* and *Bifidobacterium*; supports mucosal immunity	Human observational/interventional studies (midlife women)	Associated with restoration of bacterial taxa reported to decline during menopause	[7]
Prebiotic (resistant starch)	Resistant starch (RS)	Enhances *Faecalibacterium* and *Roseburia* abundance; increases butyrate production	Preclinical and mixed human data	Demonstrates anti-inflammatory and neuromodulatory effects; direct evidence on anxiety outcomes remains limited	[5]

One of the most promising frontiers is personalized nutrition based on gut microbiome profiling. Considering the individual variability of the microbiota, hormonal fluctuations and environmental factors, it is realistic to envisage that dietary interventions “tailored” to microbial and metabolic phenotypes could maximize efficacy. Rather than a single therapeutic pathway, current evidence supports a flexible, individualized framework informed by microbiome, metabolic, hormonal, and lifestyle characteristics.

Translational research suggests that profiling the microbiota, intestinal metabolites and barrier function may guide the selection of specific probiotic strains, fibre types or fermented food regimens. Moreover, the combination of hormonal genetics, microbial profiling and lifestyle parameters could yield predictive algorithms for prevention of perimenopausal anxiety [69].

In summary, dietary modulation of the intestinal microbiota, using probiotics, prebiotics, fermented foods, fibres and polyphenols, appears to be a biologically plausible strategy to alleviate anxiety associated with the perimenopausal period. At present, this approach should be regarded as complementary and exploratory, providing a conceptual foundation for the future development of evidence-based clinical pathways rather than an immediately implementable therapeutic protocol. From this perspective, the dietary interventions discussed do not constitute a standalone or prescriptive therapeutic pathway but should be interpreted as complementary nutritional strategies that may support and accompany conventional care in the management of perimenopausal anxiety and should therefore be considered on an individual basis, tailored to each patient’s clinical presentation, comorbidities, and concomitant treatments.

The adoption of personalized, microbiome-informed approaches opens new therapeutic possibilities, even though further high-quality clinical trials are required.

**Figure 2:**
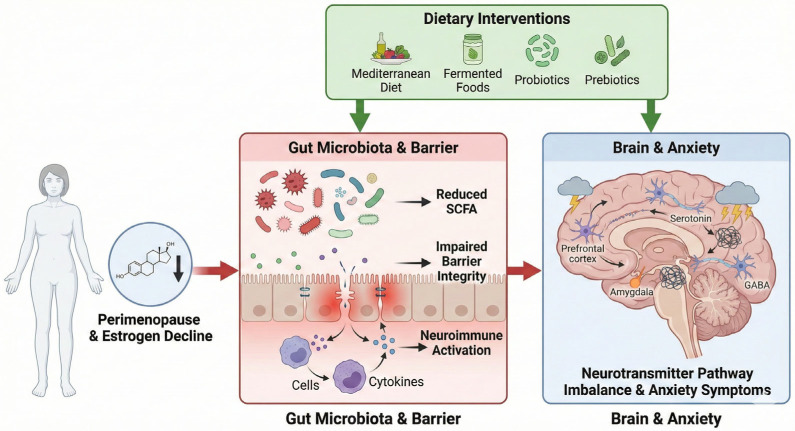
Conceptual model of the microbiota–gut–brain axis in perimenopausal anxiety. Note. Estrogen decline contributes to gut dysbiosis, reduced SCFA production, impaired barrier integrity, and neuroimmune activation, ultimately affecting neurotransmitter pathways and anxiety symptoms. Dietary interventions (Mediterranean diet, fermented foods, probiotics, and prebiotics) may help restore microbial balance and mitigate anxiety-related outcomes. Abbreviations. SCFA: Short-Chain Fatty Acids; GABA: Gamma-Aminobutyric Acid.

To visually integrate the evidence discussed in this section, Figure 2 illustrates a unified mechanistic model linking perimenopausal hormonal decline to gut microbiota alterations, neuroimmune dysregulation, and anxiety vulnerability. The figure also summarizes how dietary strategies, including Mediterranean-style patterns, fermented foods, probiotics, and prebiotics, may counteract dysbiosis and support emotional well-being during the menopausal transition.

## 7. Challenges and Future Directions

Despite growing interest in the interplay between menopausal hormonal changes, anxiety disorders, and the gut microbiota, several challenges currently limit the translation of existing evidence into clinical practice. A major limitation is the scarcity of robust data derived from randomized controlled clinical trials. Most of the existing findings are based on small cohorts, heterogeneous study populations, or preclinical models, which constrains the ability to establish causal relationships between dysbiosis, neuroendocrine pathways, and anxiety symptoms. 

Moreover, the wide variety of methodologies used to assess microbiome composition, including differences in sequencing platforms and sample-processing protocols, further complicates comparisons across studies.

In addition, the gut microbiota is highly individualized and shaped by multiple interacting factors, such as diet, lifestyle, comorbid medical condition, and medication use. This interindividual variability poses a significant challenge to the development of universally applicable microbiota-targeted interventions.

In addition, it should be explicitly acknowledged that the observed associations between gut microbiota and anxiety during perimenopause may be substantially influenced by several confounding factors. Lifestyle-related variables, including sleep quality and physical activity, medication use, coexisting medical conditions, and chronic psychosocial stress are all known to independently affect the gut–brain axis. Consequently, microbiota–anxiety correlations reported in perimenopausal women are likely to be entangled with, and potentially amplified by, these factors, complicating the interpretation of findings and limiting causal inference.

Well-designed randomized controlled trials aimed at evaluating microbiota-targeted interventions (dietary patterns, probiotics, prebiotics, synbiotics, and personalized nutrition) in women experiencing specific perimenopausal symptoms are warranted. Integrating new approaches, such as metagenomics, metabolomics, and hormonal profiling, will be essential to clarify the interactions between the microbiota, estrogen levels, and neuroimmune pathways. Personalized nutritional strategies based on microbiotic and metabolic phenotypes represent a promising frontier and may, in the future, guide tailored interventions capable of achieving meaningful clinical benefit. 

Ultimately, progress in this field will require standardized methodologies and interdisciplinary collaborations involving endocrinology, psychiatry, nutritional sciences, and microbiome research.

## 8. Conclusions

Perimenopause represents a critical neuroendocrine transition characterized by pronounced hormonal fluctuations that interact with immune, metabolic, and neuroendocrine pathways, thereby increasing vulnerability to anxiety symptoms. Accumulating evidence shows that these hormonal changes are accompanied by significant alterations in gut microbiota composition, including reduced diversity, loss of beneficial taxa, and a shift toward pro-inflammatory microbial profiles. Such dysbiosis may contribute to anxiety through multiple, interconnected mechanisms involving impaired intestinal barrier function, altered microbial metabolite production, neuroimmune activation, and dysregulation of stress-response systems.

Dietary interventions that support a healthy microbiota, such as Mediterranean-style patterns, fibre- and polyphenol-rich foods, fermented products, probiotics, and prebiotics, represent promising complementary strategies to improve emotional well-being during this transition. However, robust perimenopause-specific randomized controlled trials are still lacking, and individual variability in microbiome composition limits the ability to generalize findings.

Future research should prioritize high-quality clinical trials and integrative approaches combining microbiome, metabolic, and hormonal profiling. Personalized, microbiome-informed nutritional strategies hold promise for optimizing preventive and supportive interventions for anxiety during perimenopause.

By specifically framing perimenopause as a critical neuroendocrine window of vulnerability to anxiety and integrating hormonal dynamics, gut microbiota alterations, and dietary modulation within a unified conceptual framework, this review advances current understanding and highlights key gaps for future translational and clinical research.

## Data Availability

No new data were created or analyzed in this study. Data sharing is not applicable to this article.

## Abbreviations

The following abbreviations are used in this manuscript:

FSH	Follicle-Stimulating Hormone
LH	Luteinizing Hormone
CNS	Central Nervous System
HPG axis	Hypothalamic–Pituitary–Gonadal axis
GnRH	Gonadotropin-Releasing Hormone
NKB	Neurokinin B
Kp	Kisspeptin
DYN	Dynorphin
KNDy	Kisspeptin/Neurokinin B/Dynorphin neurons
VMS	Vasomotor Symptoms
CES-D	Center for Epidemiologic Studies Depression Scale
GSM	Genitourinary Syndrome of Menopause
MHT	Menopausal Hormone Therapy
MD	Mediterranean Diet
SCFA	Short-Chain Fatty Acids
GABA	Gamma-Aminobutyric Acid
5-HT	Serotonin
DA	Dopamine
NE	Norepinephrine
ENS	Enteric Nervous System
ANS	Autonomic Nervous System
HPA axis	Hypothalamic–Pituitary–Adrenal axis
MGBA	Microbiota–Gut–Brain Axis
GOS	Galacto-Oligosaccharides
FOS	Fructo-Oligosaccharides
RS	Resistant Starch
